# Identification of a novel recombinant polerovirus and other emergent viruses and tombusvirus-like associated RNA species associated with carrot motley dwarf disease in the United States

**DOI:** 10.3389/fmicb.2024.1430445

**Published:** 2024-07-26

**Authors:** Anna Erickson, Anneliek M. Ter Horst, Curtis R. Carlson, Bryce W. Falk, Yen-Wen Kuo

**Affiliations:** Department of Plant Pathology, University of California, Davis, CA, Unites States

**Keywords:** virus co-infection, recombinant polerovirus, emergent viruses, RNAseq, viral disease complex

## Abstract

Carrot motley dwarf (CMD) is a viral disease complex caused by co-infection of the polerovirus carrot red leaf virus with the umbraviruses carrot mottle virus or carrot mottle mimic virus, and/or a tombusvirus like associated RNA (tlaRNA), which depend on co-infection with a helper polerovirus to gain aphid transmissibility. In 2020 and 2021 carrot samples from Washington, United States (U.S.), and parsley and cilantro samples from California, U.S., exhibiting typical symptoms of CMD were submitted for diagnosis. Initial RT-PCR diagnostic assays identified the typical CMD viruses in the carrot samples, however only the umbraviruses and tlaRNAs were detected in the parsley and cilantro samples; as such, these samples were retested with another RT-PCR assay for generic polerovirus detection. Unexpectedly, the poleroviruses Torilis crimson leaf virus (TorCLV) and fennel motley virus were identified. Subsequent RNA sequencing analysis was conducted to confirm these results and look for other emergent viruses. In addition to confirming the diagnostic results, the recently described polerovirus Foeniculum vulgare polerovirus, the umbraviruses Pastinaca umbravirus 1 and wild carrot mottle virus, and the tlaRNA Arracacha latent virus E associated RNA were identified, making this the first report of these viruses and tlaRNA in the U.S. Using phylogenetic and pairwise identity comparisons and RDP4 recombination analyses, we also identified a putative novel polerovirus, for which we propose the name parsley polerovirus, that appears to be a recombinant between carrot polerovirus 1, sharing 92% amino acid (aa) identity with the RNA dependent RNA polymerase in the 5′ gene block, and TorCLV, sharing >98% aa identity with the capsid protein in the 3 gene block. This work adds to the growing list of polerovirus species exhibiting recombination between the 5′ and 3′ gene blocks, and highlights the unique, variable, and dynamic associations that can occur in polerovirus, umbravirus, and tlaRNA disease complexes.

## Introduction

Carrot motley dwarf (CMD) is a viral disease complex caused by co-infection of a polerovirus in combination with an umbravirus and/or one of several subviral agents referred to as tombusvirus-like associated RNAs (tlaRNAs), all of which have positive sense, single stranded RNA (+ssRNA) genomes (Murant et al., 1985; Watson et al., 1998; Syller, 2003; Taliansky and Robinson, 2003; Huang et al., 2005; Campbell et al., 2020; LaTourrette et al., 2021). Viruses and tlaRNAs historically known to be associated with CMD include the polerovirus carrot red leaf virus (CtRLV), the umbraviruses carrot mottle virus (CMoV) and carrot mottle mimic virus (CMoMV), and a multitude of tlaRNAs: carrot red leaf virus associated RNAs (CtRLVaRNAs) a8, a25, alpha, beta, gamma, sigma, SN, and HK (Watson et al., 1964; Murant and Roberts, 1979; Waterhouse and Murant, 1981; Murant et al., 1985; Gibbs et al., 1996a,b; Campbell et al., 2020; Yoshida, 2020). In addition to carrots (*Daucus carota*), CMD viruses can infect a variety of other plants within the Apiaceae family including coriander/cilantro (*Coriandrum sativum*), chervil (*Anthriscus cerefolium*), cumin (*Cuminum cyminum*), and parsley (*Petroselinum crispum*) (Watson and Serjeant, 1964; Tang et al., 2009; Yoshida, 2020). These CMD viruses and tlaRNAs are vectored by the willow-carrot aphid (*Cavariella aegopodii*) and can be found throughout the world wherever carrots are grown (Watson and Falk, 1994). While sporadic, outbreaks of CMD can cause severe losses in carrot crops, and the severity of symptoms is highly dependent on the carrot cultivar, plant age, environmental conditions (cool temperatures and low-light conditions are more conducive) and the number of these viruses and tlaRNAs co-infecting the plant. With the production value of carrots in the U.S. reaching $1.82 billion in 2023, outbreaks of CMD can have significant economic impacts (United States Department of Agriculture-National Agricultural Statistics Services [USDA-NASS], 2024).

Experimental inoculations of CtRLV, CMoV or CMoMV, and CtRLVaRNAs have shown symptoms caused by individual infections of these viruses and tlaRNAs are relatively mild to absent, whereas those caused by double infections of CtRLV with either

more severe, and infections of all three of these agents produce the most severe symptoms hallmarked by vibrant yellow to red mottled discoloration of leaf tips and margins, and severe stunting (Watson et al., 1964; Watson and Serjeant, 1964; Watson and Falk, 1994; Yoshida, 2020; Erickson and Falk, 2023). However, single infections of umbraviruses or CtRLVaRNAs have not been observed in the field (Elnagar and Murant, 1978a,b; Waterhouse and Murant, 1983). This is due to the unique nature of these particular disease complexes, wherein, despite each virus being able to replicate autonomously, umbraviruses and tlaRNAs both rely on interactions with a compatible co-infecting polerovirus to gain necessary functions.

Poleroviruses are completely autonomous, phloem-limited viruses that encode their own capsid proteins which, in combination with the P3a protein, allow them to move systemically within a plant and be transmitted between hosts by aphid vectors in a persistent, non-propagative manner (Rochow, 1972; Cilia et al., 2014; Delfosse et al., 2021). While umbraviruses can move both systemically in the phloem and locally between mesophyll cells, and can be mechanically transmitted—though this is not known to be a primary means of transmission in the field—they do not encode their own capsid proteins and are thus dependent on co-infection with a polerovirus, wherein their genomic RNAs can be transcapsidated by polerovirus capsid proteins in order to become aphid transmissible (Murant et al., 1969; Elnagar and Murant, 1978a,b; Waterhouse and Murant, 1983; Taliansky and Robinson, 2003). TlaRNAs only encode an RNA-dependent RNA polymerase, and are therefore dependent on a co-infecting polerovirus and/or umbravirus for within host movement and to gain aphid or mechanical transmissibility (Campbell et al., 2020; Erickson and Falk, 2023).

In the spring and summer of 2020, our lab received for diagnosis carrot samples collected in western (Jefferson county) and central (Grant county) Washington, U.S., curly and flat leaf parsley samples collected in Ventura county, California, U.S., and a cilantro sample collected in Yolo, county California, U.S., exhibiting typical CMD symptoms. We tested these samples for CtRLV, CMoV/CMoMV, and CtRLVaRNAs using previously described RT-PCR based assays (Vercruysse et al., 2000; Campbell et al., 2020). The carrot samples tested positive for all of the typical CMD associated viruses and tlaRNAs. However, while the parsley and cilantro samples tested positive for CMoV/CMoMV and/or CtRLVaRNAs, none tested positive for CtRLV. Given the dependency of umbraviruses and tlaRNAs on a co-infecting polerovirus for aphid transmission, we retested these samples using degenerate primers for polerovirus detection, as described (Lotos et al., 2014), and obtained amplicons of the expected size. Sanger sequencing of these PCR products identified the unknown polerovirus as Torilis crimson leaf virus (TorCLV) (GenBank accession: LT595017.1).

These results prompted us to conduct an exploratory RNA sequencing (RNAseq) based analysis of these samples, along with the carrot samples from Washington, and additional parsley samples (Ventura county, California) and a cilantro sample (Yolo county, California) collected in 2021 to confirm the presence of TorCLV and determine if other emergent polerovirus, umbravirus, and tlaRNA species were present. In this study we describe the detection of the classically known CMD-associated viruses and tlaRNAs, along with several recently described virus and tlaRNA species—two poleroviruses, Torilis crimson leaf virus (TorCLV) and Foeniculum vulgare polerovirus (FvPV), two umbraviruses, Pastinaca umbravirus 1 (PasUV1) and wild carrot mottle virus (WCMoV), and one tlaRNA species, arracacha latent virus E associated RNA (ALVEaRNA)—not previously detected in the U.S. or in association with CMD, and one novel polerovirus species that appears to be a recombinant of TorCLV and another recently identified polerovirus, carrot polerovirus 1 (CaPV1; accession: OP886450.1). Not only does this study shed light on the complexity of known and emergent viruses and tlaRNAs that may contribute to CMD in parsley and cilantro plants, it also highlights the ongoing utility of using high throughput sequencing (HTS) technologies for the discovery and characterization of emergent, and potentially economically important, plant viruses and subviral agents.

## Materials and methods

### Plant material and virus diagnostics

**FIGURE 1:**
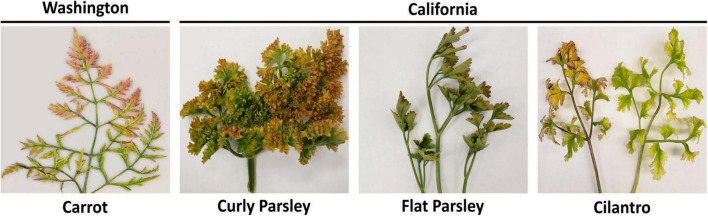
Carrot, parsley, and cilantro samples exhibiting typical symptoms of carrot motley dwarf disease. Depicted are representative carrot, flat and curly leaf parsley, and cilantro samples exhibiting typical symptoms of carrot motley dwarf disease, hallmarked by the yellow, orange, and red leaf discoloration.

Descriptive details about the carrot, parsley, and cilantro samples used for RNAseq analysis in this study, including the library IDs and number of sequencing reads obtained per sample, can be found in Table 1. From the carrot, parsley, and cilantro sample sets submitted to our lab for diagnosis, individual samples exhibiting the most obvious symptoms of CMD (yellow or red discoloration of leaf tips and margins) were selected for testing (Figure 1). A portion of each sample was cut into small pieces and the tissue was split into two aliquots, one of which was flash frozen in liquid nitrogen and stored at −80°C and the other vacuum dried for 4 days then stored at −20°C for future use.

**TABLE 1:** Library IDs, number of clean reads per library, and descriptive information for the plant samples used in this study.

Library ID	No. clean reads	Host	Sample group	Location	Collection year	Notes
AECMD_01	16400000	Cilantro	H1	Davis, CA	2021	Grown from seed in growth chamber
AECMD_02	14600000	Carrot	H2			
AECMD_03	14900000	Curly parsley	H3			
AECMD_04	13800000	Flat parsley	H4			
AECMD_05	15600000	Flat parsley	1	Ventura Co., CA	2020	Same field/planting, different blocks
AECMD_06	15800000					
AECMD_07	13400000					
AECMD_08	13200000	Curly parsley	2	Ventura Co., CA	2020	
AECMD_09	19600000					
AECMD_10	12700000					
AECMD_11	12200000	Flat parsley	3	Ventura Co., CA	2021	No additional information provided
AECMD_12	10800000					
AECMD_13	13800000					
AECMD_14	12500000	Curly parsley	4	Ventura Co., CA	2021	Overwintered samples from same field as groups 1 and 2
AECMD_15	15000000					
AECMD_16	11200000	Flat parsley				
AECMD_17	14400000	Curly parsley	5	Ventura Co., CA	2021	
;AECMD_18	15900000					
AECMD_19	11200000					
AECMD_20	12400000	Curly parsley	6	Monterey Co., CA	2021	No additional information provided
AECMD_21	14900000					
AECMD_22	15900000					
AECMD_23	10700000	Cilantro	7	Yolo, Co., CA	2020	From resident yard; *Dysaphis apifolia* aphids present
AECMD_24	12200000	Cilantro	8	Yolo, Co., CA	2021	From UC Davis student farm
AECMD_25	15200000	Carrot	9	Jefferson Co., WA	2020	Cage grown; red variety
AECMD_26	15200000					
AECMD_27	14900000					
AECMD_28	12900000	Carrot	10	Jefferson Co., WA	2020	Cage grown; variety unspecified
AECMD_29	14600000					
AECMD_30	11700000					
AECMD_31	15800000	Carrot	11	Jefferson Co., WA	2020	Field grown; red variety
AECMD_32	13500000					
AECMD_33	8900000					
AECMD_34	9700000	Carrot	12	Grant Co., WA	2020	Field grown; variety
AECMD_35	10900000					
AECMD_36	11500000					

The remaining sample tissues were pooled according to location of origin, and total RNA was extracted using TRIzol™ Reagent (Invitrogen) and used as template for cDNA synthesis using the SuperScript™ III Reverse Transcriptase kit (Invitrogen), according to the manufacturer’s protocols. RT-PCR was performed using the GoTaq^®^ Flexi DNA Polymerase kit (Promega) in a 25 μl reaction containing 5 μl of 5X Green GoTaq^®^ Flexi Buffer, 1.5 μl of 25 mM MgCl_2_, 1.25 μl of each primer (10 μM), 0.5 μl of 10 mM dNTPs, 0.125 μl of Taq polymerase, 2 μl of cDNA, and 14.375 μl of nuclease free water. The primers and thermocycling conditions used for CMoV and CtRLV are detailed in Vercruysse et al., 2000 and Campbell et al., 2020, respectively. RT-PCR products were visualized by 2% agarose gel electrophoresis, and RT-PCR products of the expected molecular weight were purified using the NucleoSpin Gel and PCR Clean-up kit (Takara Bio) and sent for Sanger sequencing (genewiz.com) Samples that tested negative for CtRLV were subjected to a subsequent RT-PCR assay designed for the generic detection of poleroviruses, as described (Lotos et al., 2014). All primers used in this study are listed in Supplementary Table 1.

### Sample preparation for RNA sequencing (RNA-seq)

Tissue from the individual plant samples, as well as from healthy flat and curly leaf parsley, cilantro, and carrot plants grown from seed in a growth chamber, were used for total RNA extraction using the RNeasy Plant Mini Kit (Qiagen) according to the manufacturer’s instructions. RNA samples were DNase treated with RQ1 RNase-Free DNase (Promega) and cleaned using the “RNA Cleanup” protocol from the same extraction kit. The concentration and integrity of RNA samples were checked using the Qubit 4 Fluorometer (ThermoFisher Scientific) and the Experion™ Automated Electrophoresis System bioanalyzer. RNA samples were submitted to the DNA Technologies and Expression Analysis Core Laboratory at the UC Davis genome center for ribodepletion, library preparation, and transcriptome sequencing on the NovaSeq 6000 platform (Illumina). Aliquots of each RNA sample were retained for RT-PCR validation of viral sequences obtained by RNAseq.

### Bioinformatic analysis

The returned raw read data was processed and analyzed according to a previously established pipeline for detecting viral RNAs in plant samples (Ter Horst et al., 2023). In brief, the raw read data was quality checked using FastQC v0.11.9 (Andrews, 2010), adapter sequences and low-quality reads were removed using Trimmomatic v0.40 (Bolger et al., 2014), and the clean read data was quality checked again. Clean reads were assembled with MEGAHIT v1.02 (Li et al., 2015) using default settings with a minimum contig length of 200 bp. Prodigal v2.6.3 (Hyatt et al., 2010) was used to predict protein coding sequences from the assembled contiguous sequences (contigs), the output of which was then subjected to analysis with HMMR v3.3.2 (Finn et al., 2011) to search for viral RNA dependent RNA polymerase (RdRp)-like sequences using the protocols and HMM profiles established by Wolf et al. (2018), with the default *E*-value cutoff. Returned RdRp-like contigs were compared against the NCBI nucleotide (nt) database using BLASTn, and predicted protein coding sequences were used as query in a search against the NCBI non-redundant (nr) protein database using BLASTp (accessed September 2022). The raw read data was uploaded to the NCBI database under BioProject accession PRJNA1099309.

The BLASTn and BLASTp results were then manually curated. Sequencing reads were mapped back against selected virus contigs of interest using Samtools v1.11 software, and coverage tables were generated with coverM v0.6.1 software using the mean method to determine the mean number of aligned reads that overlapped each position of the contig. Viral contigs > 1000 nt in length, represented by > 1% of total virus reads in the sample group, having > 100x average genome coverage when mapped back to reference sequences, and sharing homology with putative poleroviruses, umbraviruses, and tlaRNAs were selected for manual inspection. In some of the retrieved viral contigs, contaminating host sequences were found. To confirm these contaminating host sequences were artifactual, a representative contig was loaded into the integrative genomics viewer (IGV) (Robinson et al., 2011), then manually inspected for reads spanning the junction between host and viral sequences, of which none were found (Supplementary Figure 1). Contigs of interest were aligned with reference virus genomes obtained from the NCBI GenBank database that were indicated in the BLASTn and BLASTp outputs using SnapGene software.^1^ Viral contigs that did not align with the selected viruses or tlaRNAs were subjected to BLASTn and/or BLASTx analysis to identify the closest virus relative.

### RT-PCR and nanopore sequencing validation

Specific primers were designed using SnapGene v6.1 software^1^ to amplify nearly full length genomic sequences of most of the viruses and tlaRNAs identified (CtRLV, TorCLV, FvPV, PaPV, CMoV, CMoMV, WCMoV, PasUV1, CtRLVaRNAs alpha, a25, sigma, and ALVEaRNA) (Supplementary Figure 2). Most of the tlaRNA sequences obtained by RNAseq shared extremely high sequence homology which complicated primer design for specific isolates, as such primers were designed to detect any of these tlaRNAs. The CtRLVaRNA sigma and arracacha latent virus associated RNA (ALVEaRNA) were dissimilar enough that specific primers could be designed.

Aliquots of the same RNA samples submitted for RNA-seq analysis were used as templates for cDNA synthesis using the SuperScript™ IV Reverse Transcriptase kit (Invitrogen). PCR was performed using CloneAmp HiFi PCR Premix (Takara) in 25 μl reactions containing 12.5 μl of premix, 1 μl of each primer (10 μM), and 1 μl of cDNA; the thermocycling conditions were as follows: 95°C for 1 min, followed by 40 cycles of 98°C for 10 s, 50–55°C (Supplementary Table 1), 72°C for 40–70 s (Supplementary Table 1), and a final extension at 72°C for 5 min. PCR products were gel purified, cloned into the pCR-XL-2-TOPO vector (Invitrogen), and submitted for whole plasmid Nanopore sequencing (Eurofins Genomics), to verify the sequences obtained by RNAseq. Details of the cloned sequences submitted for nanopore sequencing can be found in Supplementary Table 2.

### 5′ and 3′ RACE to obtain full length virus genome sequences of PaPV and FvPV

To obtain full length genome sequences of the novel recombinant polerovirus identified in this study, along with FvPV for which only partial genome sequences were available in the GenBank database, 5′ and 3′ RACE (rapid amplification of cDNA ends) PCR was performed. For 5′ RACE, fresh RNA extracted from stored tissue samples was reversed transcribed using SuperScript IV Reverse Transcriptase (Invitrogen) with gene specific primers (GSPs) positioned near the 5′ end of the viral genomes. The resulting first strand cDNA was purified using the DNA Clean and Concentrator Kit-5 (Zymo), then C-tailed using Terminal Transferase (New England Biolabs) as per the manufacturer’s instructions. Subsequently, nested PCRs with GSPs and 5′ adapter primers (Aps) were done in a 25 μl reaction mix containing 12.5 μl of 2x PrimeSTAR GXL premix (Takarabio.com), 1 μl of each primer (10 μM), using the following cycling conditions: 35 cycles of 98°C for 10 s, 60°C for 15 s, and 98°C for 2 m. For 3′ RACE, since poleroviruses lack poly-A tails, freshly extracted RNA was polyadenylated using E. coli Poly(A) Polymerase (New England Biolabs) as per the manufacturer’s instructions, prior to RT-PCR. After purifying the A-tailed RNA using the RNeasy Plant Mini Kit (Qiagen), the RT-PCR was performed as described for 5′ RACE, but using 3′ Aps and GSPs. Finally, PCR products were cloned and sent for Sanger sequencing. Subsequently, the full length sequences of each virus were also cloned and confirmed by Sanger sequencing.

### Phylogenetic classification and percent pairwise identity analysis

To determine the relative taxonomic positions of the poleroviruses, umbraviruses, and tlaRNAs identified in this study to those of previously described viruses and tlaRNAs, phylogenetic analyses were performed. According to the International Committee on Taxonomy of Viruses (ICTV), the species demarcation for poleroviruses is a > 10% difference in shared amino acid (aa) sequence identity for any of the encoded gene products (Walker et al., 2022). Umbravirus species are demarcated by < 70% shared nt sequence identity of the genome. As they are currently unclassified, there is no set species demarcation criteria for tlaRNAs. For the poleroviruses, we used near to full length translated aa sequences of the P1-P2 fusion protein (RdRp) and the P3 protein (CP), for the umbraviruses we used nearly full length genome sequences, and for the tlaRNAs we used nearly full length translated aa sequences of the combined P1a+P1b (RdRp) predicted readthrough protein. The nt or aa sequences from the virus and subviral agents identified in this study, along with those of reference isolates, were aligned with the MUSCLE algorithm using MEGA11 software (Tamura et al., 2021). Maximum likelihood (ML) phylogenetic analyses using 1000 bootstrap replicates were performed after using the MEGA11 model selection tool to identify the most appropriate substitution models (Supplementary Table 3). Pairwise identity comparisons were calculated using SDT v1.2 (Muhire et al., 2014). The phylogenetic tree and identity matrices were edited using Inkscape v1.2 software.^2^ Accession numbers for the representative virus isolate sequences used for these analyses are listed in Table 4.

**TABLE 2:** Contig counts and lengths, and sample origin of each plant virus species identified by BLASTx analysis.

Family	Genus	Species	No. contigs	Contig lengths	Sample group(s)
*Solemoviridae*	*Polerovirus*	Torilis crimson leaf virus (TorCLV)	43	240–8329	1–8
		carrot red leaf virus (CtRLV)	13	281–6630	9–12
		wild carrot red leaf virus (WCtRLV)	12	548–6313	8
*Tombusviridae*	*Umbravirus*	carrot mottle virus (CMoV)	48	282–5466	2–4, 9–11
		carrot mottle mimic virus (CMoMV)	21	1260–5117	1–5, 7–11
		wild carrot mottle virus (WCMoV)	16	398–6263	2–4, 9–11
		parsley mottle virus*	1	719	1–12
		parsley mottle mimic virus*	3	253–360	2, 4
		tobacco bushy top virus* (TBTV)	1	574	2–7
Unclassified	tombusvirus-like associated RNA (tlaRNA)	arracacha latent virus E aRNA (ALVEaRNA)	8	507–4023	2, 6, 11
		CtRLVaRNA a8	1	1284	9
		CtRLVaRNA a25	8	458–5910	10–12
		CtRLVaRNA alpha	4	310–919	9, 12
		CtRLVaRNA gamma	3	2009–2493	9, 12
		CtRLVaRNA sigma	8	281–3411	2, 6
		CtRLVaRNA SH	5	496–3333	9, 10, 12
		CtRLVaRNA HK2	3	314–870	11, 12
		tlaRNA POR19SW	1	859	6
*Rhabdoviridae*	*Cytorhabdovirus*	alfalfa dwarf virus	1	566	4, 5
		raspberry vein chlorosis virus	1	859	4, 5, 7
		suaeda salsa virus 1	4	376–8330	8, 9
*Closteroviridae*	*Crinivirus*	beet pseudoyellows virus	3	7088–7924	7–8
*Potyviridae*	*Potyvirus*	carrot thin leaf virus	1	9711	12
		watermelon mosaic virus	1	12645	8
*Secoviridae*	*Waikavirus*	bellflower vein chlorosis virus	3	310–12119	12
		red clover associated virus	6	3242–5831	3, 4
	*Torradovirus*	carrot torradovirus 1	3	4581–8941	12
*Partitiviridae*	*Alphapartitivirus*	carrot cryptic virus	1	2170	11
	*Betapartitivirus*	dill cryptic virus 2	4	2344–2520	11
	Unclassified	persimmon cryptic virus	4	655–1744	11
*Bromoviridae*	*Alfamovirus*	alfalfa mosaic virus	1	6432	10
	*Cucumovirus*	cucumber mosaic virus	1	327	11
	*Ilarvirus*	raphanus latent virus	1	468	3
*Totiviridae*	*Totivirus*	black raspberry virus F	3	5122–7012	12
Unclassified	Unclassified	red clover RNA virus 1	4	6124–8209	11, 12

*These umbraviruses were excluded from downstream analysis as they were very poorly represented in the dataset i.e., few contigs of short length were retrieved, and were represented by < 1% of total virus reads, and/or had < 100x genome coverage, relative to other retrieved polerovirus, umbravirus, and tlaRNA sequences.

**TABLE 3:** The average genome coverage, number, and percentage of sequencing reads that mapped back to potential CMD associated virus contigs identified by RNAseq.

Viruses		Host	Sample group	No. virus reads	% of total virus reads	Avg. genome coverage
Poleroviruses	TorCLV	Parsley	1	6226019	13.9%	1025
			2	5709876	12.5%	1005
			3	6009999	16.3%	1508
			4	15449038	39.9%	3831
			5	14503124	34.9%	2670
			6	2610644	6.0%	471
	CtRLV	Carrot	9	1093563	2.4%	682
			10	1701116	4.3%	1163
			11	713196	1.9%	492
			12	3651518	11.4%	3293
	WCtRLV	Cilantro	8	2800544	3.0%	6207
Umbraviruses	CMoV	Parsley	2	3121520	6.9%	823
			3	1565616	4.3%	508
			4	1502702	3.9%	405
			5	1427207	3.4%	341
		Carrot	9	2189317	4.8%	613
			10	2257445	5.8%	636
			11	1019243	2.7%	334
	CMoMV	Parsley	3	591679	1.6%	395
			4	593670	1.5%	313
		Carrot	9	1066909	2.4%	539
			10	1520888	3.9%	948
			11	1801653	4.7%	1307
	WCMoV	Parsley	1	2218037	5.0%	984
			2	2796696	6.1%	1529
		Carrot	9	2039937	4.5%	1123
			10	2272673	5.8%	1473
			11	3871682	10.1%	2640
			12	1388004	4.3%	1419
	parsley mottle virus*	Parsley	1	2580	< 1%	88
			2	2862	< 1%	102
			3	4797	< 1%	192
			4	2231	< 1%	85
			5	2793	< 1%	105
			6	2540	< 1%	55
		Cilantro	7	1386	< 1%	127
	parsley mottle mimic virus*	Parsley	2	1725	< 1%	87
			4	4	< 1%	0.1
	TBTV*	Parsley	2	388	< 1%	26
			3	156	< 1%	14
			4	259	< 1%	22
			5	88	< 1%	5
			6	58	< 1%	4
		Cilantro	7	80	< 1%	18
tlaRNAs	ALVEaRNA	Carrot	11	3417453	8.9%	8808
	CtRLVaRNA a8	Carrot	9	933920	2.1%	34168
	CtRLVaRNA a25	Carrot	9	3057812	6.8%	3393
			10	1584673	4.0%	2780
			11	2104029	5.5%	4491
			12	1951914	6.1%	7335
	CtRLVaRNA alpha	Carrot	9	557056	1.2%	8014
	CtRLVaRNA gamma	Carrot	11	2104029	5.5%	3985
	CtRLVaRNA sigma	Parsley	2	5486414	12.1%	14800
	CtRLVaRNA SH	Carrot	10	605490	1.5%	2525
			12	2020040	6.3%	16258

*These umbraviruses were excluded from downstream analysis as they were very poorly represented in the dataset i.e. few contigs of short length were retrieved, and were represented by < 1% of total virus reads, and/or had < 100x genome coverage, relative to other retrieved polerovirus, umbravirus, and tlaRNA sequences.

**TABLE 4:** Summary of poleroviruses, umbraviruses, and tlaRNAs identified in each plant sample by RNAseq and validated by RT-PCR and nanopore and/or Sanger sequencing.

Virus		Accession(s)	Host	Identified by RNAseq	Confirmed by RT-PCR and sequencing
Poleroviruses	TorCLV	PP888040	Parsley	6–19, 22	6, 8–22
		PP888041	Cilantro	23	23
	PaPV	PP683457; PP683458	Parsley	8, 9, 11, 13, 14, 20, 22	8–15, 18, 20
	FvPV	PP683459	Cilantro	23	23
	CtRLV	PP888039	Carrot	26–36	26–36
Umbraviruses	CMoV	PP766558	Carrot	26–29, 32,33, 35	26–29, 32,33, 35
	CMoMV	PP766560	Parsley	9–15, 20, 22	9–15, 20, 22,
		PP766559	Carrot	26–29, 31–33	26–29, 31–33
	WCMoV	PP766561	Parsley	8	8
		PP766562	Carrot	26–34, 36	26–34, 36
	PasUV1	PP766563	Parsley	8–15, 18, 20–22	8–15, 18, 20
		PP766564	Cilantro	23	not amplified
tlaRNAs	CtRLVaRNA a25	PP766566	Cilantro	24	24
		PP766565	Carrot	28, 29, 31	isolated/sequenced from pooled sample
	CtRLVaRNA alpha	PP766570	Carrot	26, 29	isolated/sequenced from pooled sample
	CtRLVaRNA beta	PP766567	Carrot	33, 34	not isolated
	CtRLVaRNA gamma	PP766568	Carrot	33	not isolated
	CtRLVaRNA sigma	PP766569	Parsley	8–10, 22	8–10, 22
	ALVEaRNA	PP766571	Parsley	9, 10, 20, 22	9, 10, 20, 22
		PP766572	Carrot	32	32

### Recombination analysis

Full length sequences (confirmed by Sanger sequencing) of two isolates of the novel recombinant polerovirus identified in this study, along with the reference genomes for the two most closely related poleroviruses identified by BLASTn—TorCLV and CaPV1—were aligned using MEGA11, then analyzed for recombination events using the RDP4 software package (Martin et al., 2015).

## Results

### Detection of CMD viruses and tlaRNAs in plant samples submitted for diagnosis

**FIGURE 2:**
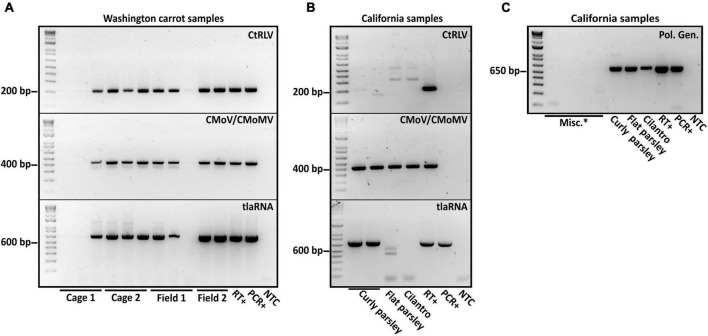
Results of diagnostic RT-PCR assays for the detection of the polerovirus carrot red leaf virus (CtRLV), the umbraviruses carrot mottle virus (CMoV) and carrot mottle mimic virus (CMoMV) and tombusvirus-like associated RNAs (tlaRNAs) associated with carrot motley dwarf disease in panel **(A)** pooled carrot samples and **(B)** pooled parsley samples, and an individual cilantro sample. The expected product sizes for CtRLV, CMoV/CMoMV, and tlaRNAs, respectively, are 211, 408, and 650 bp. PCR products from a separate RT-PCR diagnostic assay for the generic detection of poleroviruses (product size: 593 bp) are depicted in panel. **(C)** Labels in the upper right corner of the gels indicate the viruses being tested for, labels above the gels indicate the state of origin, and labels below indicate the sample or sample group. RT+, reverse transcription positive control; PCR+, positive control for PCR; NTC, no template control; Misc.*: barley samples that had also been submitted for diagnosis of potential polerovirus infection.

After performing initial RT-PCR-based diagnostic assays (Vercruysse et al., 2000; Campbell et al., 2020) on flat and curly leaf parsley, carrot, and cilantro samples exhibiting typical symptoms of CMD (Figure 1), we found that all of the known CMD-associated viruses and tlaRNAs were present in the carrot samples (Figure 2A). Unexpectedly, only umbraviruses (CMoV and/or CMoMV) and tlaRNAs were detected in the parsley and cilantro samples, in the absence of the known helper polerovirus, CtRLV (Figure 2B). Given the dependence of umbraviruses and tlaRNAs on a coinfecting polerovirus for aphid transmission between hosts, we suspected the presence of a different polerovirus. After retesting these samples using a different RT-PCR based assay for generic polerovirus detection (Lotos et al., 2014), we obtained amplicons of the expected size (∼600 bp), which we submitted for Sanger sequencing. BLASTn analysis showed the sequence shared 95.7% nt sequence identity with a recently deposited polerovirus sequence, Torilis crimson leaf virus (TorCLV; accession LT595016.1). Additional parsley and cilantro samples received in the following year were tested using this assay; the parsley samples again tested positive for TorCLV in various combinations with CMoV/CMoMV and/or CtRLVaRNAs, however, the amplicon sequence from the cilantro sample collected in 2021 was found to share 89.8% identity with a different polerovirus, Fennel motley virus (FMV; accession LT595018.1). Given these results we selected a subset of these samples for RNA sequencing (RNAseq) to confirm the presence of the newly described viruses and tlaRNAs in these samples and determine if other new or recently discovered umbraviruses and/or tlaRNAs were present; several of the Washington carrot samples were also included.

### General summary of RNA sequencing results

In total 18 parsley (seven flat leaf and 11 curly leaf), two cilantro, and 12 carrot samples were selected for RNA sequencing. Descriptions of the samples submitted for RNAseq analysis—including the sample group, collection year, location of origin, library IDs, and clean sequencing reads obtained for each library—are detailed in Table 1.

After removing adapter sequences and low quality reads, 8.9 to 19.6 million paired end reads, approximately 150 (bp) in length were returned. In total, 545 predicted plant viral contigs represented by 10 families, 15 genera, and 35 species were assembled, ranging in length from 240 to 14,036 nt (Table 2). A 338 mycovirus, 36 arthropod virus, and four virus contigs that did not fall under any of these categories were also retrieved (Supplementary Tables 5, 6). Among the putative virus contigs returned, polerovirus, umbravirus, and tlaRNA contigs predominated. Virus contigs > 1000 nt long and tlaRNA contigs > 500 nt, represented by > 1% of viral reads, and having greater than 100x average genome coverage were selected for further analysis. Table 3 details the number and percentage of sequencing reads that mapped back to the polerovirus, umbravirus, and tlaRNAs contigs, along with their average genome coverages.

### Polerovirus sequence identification

In total three polerovirus sequences were identified by RNAseq analysis: CtRLV, TorCLV, and wild carrot red leaf virus (WCtRLV) (Tables 2, 3). CtRLV contigs covering essentially the entire length of the ∼5.7 kb reference genome and sharing high percent nt identity (> 98%) with CtRLV accessions (LC434062.1, LC434063.1, LC434061.1, LC434064.1) in the NCBI GenBank database, were present in 11 of the 12 sequenced carrot samples, but none of the tested parsley or cilantro samples. TorCLV was present in 16 out of the 18 tested parsley samples and in the cilantro sample collected in 2020, with several contigs covering nearly 100% of the ∼5.6 kb genome and sharing > 95% identity with the single reference isolate in the GenBank database (LT615235.1). TorCLV was not found in the cilantro sample collected in 2021 or in any of the tested carrot samples. Together, these results support our results from the initial diagnostic assays.

Upon closer analysis of the polerovirus sequences identified as WCtRLV in the RNAseq data, we encountered unexpected results. According to manual BLASTn analysis of each of these contigs, none were found to actually be WCtRLV. In the cilantro sample collected in 2021, which was thought to have FMV according to the initial diagnostic assays, the single ∼5.1 kb polerovirus contig present was identified as another recently described polerovirus, *Foeniculum* vulgare polerovirus (FvPV; BK059375.1), sharing 94% identity with the reference isolate. The sequence of the FvPV isolate found in this study was slightly longer than that of the partial genome sequence uploaded for the reference isolate (∼4.3 kb), so we performed RACE to obtain the full length genome sequence. FvPV sequences were found in no other samples.

Partial to nearly full length contigs misidentified as WCtRLV, ranging in length from ∼1 kb to ∼5.2 kb, were also retrieved from seven of the eighteen parsley samples. Among these, the top three results retrieved from manual BLASTn analysis of the two longest contigs (∼5.1 and ∼ 5.2 kb), retrieved from two separate parsley samples—were CaPV1, *Trachyspermum ammi* polerovirus (TaPV), and TorCLV. Despite the contigs being close to the expected full length of a typical polerovirus genome (∼5.7 kb), all three results exhibited somewhat low query coverage scores (CaPV1: 78% - 79%; TaPV: 79% - 81%; TorCLV: 70% - 85%) and the percent shared identity scores were below the species demarcation for poleroviruses (CaPV1: 88.98% - 89.53%; TaPV: 86.24% - 86.42%; TorCLV: 84.47% - 87.62%).

**FIGURE 3:**
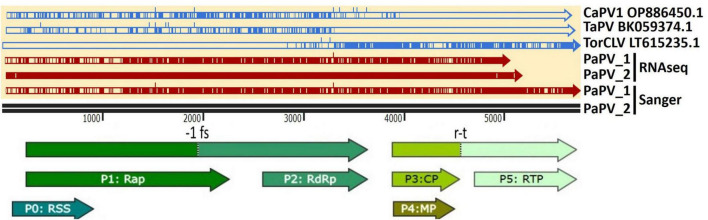
Alignments of the putative recombinant polerovirus sequences obtained in this study by RNAseq and confirmed by Sanger sequencing (colored red) with the potential parental CaPV1, TaPV, and TorCLV reference sequences (colored blue). The base reference sequence used was that of PaPV isolate 2. The double black line is a size marker for the aligned sequences, and the arrows beneath the line indicate the predicted open reading frame (ORF) translations. CaPV1, carrot polerovirus 1; TaPV, trachyspermum ammi polerovirus; TorCLV, Torilis crimson leaf virus; RSS, RNA interference silencing suppressor; Rap, replication associated protein; -1fs, -1 frameshifting site that enables P1 and P2 to be translated as a fusion protein; RdRp, RNA dependent RNA polymerase; CP, capsid protein; MP, movement protein; RTP, readthrough protein; r-t, ribosomal readthrough site that allows P3 and P5 to be translated as a fusion protein.

Visual assessment of alignments of these contigs with the CaPV1, TaPV, and TorCLV reference sequences showed that the first two thirds (∼3.5 kb) of the genome, covering the 5′ gene blocks (ORFs 0, 1, and 2), aligned more closely with CaPV and TaPV, whereas the last third of the genome (∼2.4 kb), covering the 3′ gene blocks (ORFs 3, 3a, 4, and 5), aligned more closely with TorCLV, with ∼650 bp of sequence overlap between the three reference virus sequences spanning the last ∼600 bp of ORF2 and ∼200 bp of the intergenic region (Figure 3). Given these results we suspected these contigs represented a putative new recombinant virus related to both TorCLV and either CaPV1 or TaPV. To confirm that these virus sequences were real, and not artifactual assemblies, nearly full length sequences were RT-PCR amplified from two separate parsley samples and Sanger sequenced. RACE was performed to obtain two full length (5,741 nt) isolates of this putative new polerovirus. We have chosen to tentatively name this new polerovirus parsley polerovirus (PaPV), and refer to the separate isolates as PaPV_1 and PaPV_2, which have been deposited in the NCBI database under accessions PP683457 and PP683458.

### Phylogenetic and pairwise identity comparisons of identified polerovirus sequences

The species demarcation for poleroviruses is a > 10% difference in aa sequence identity of any of the six ORF encoded proteins. To compare the phylogenetic and sequence similarity relationships of the poleroviruses identified in this study, we constructed maximum likelihood phylogenetic trees and percent shared identity matrices using translated aa sequences of the near to full length P1-P2 (RdRp) protein coding sequences from the 5′ gene block and the P3 (CP) protein coding sequence from the 3′ gene block. The cereal infecting polerovirus, Barley virus G (BVG), was used as the outgroup.

**FIGURE 4:**
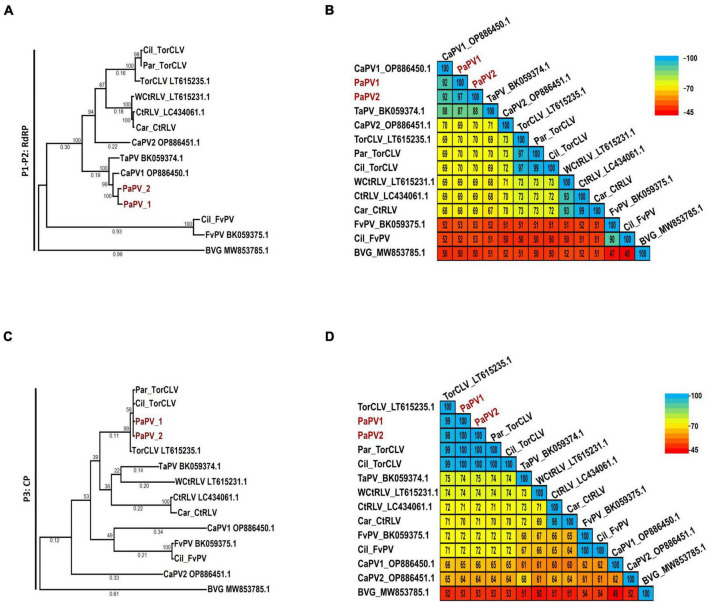
Phylogenetic trees and average percent pairwise matrices depicting relationships of the polerovirus sequences found in this study. Depicted are maximum likelihood phylogenetic trees (**A** and **C**) and pairwise identity matrices (**B** and **D**) constructed using the translated amino acid sequences of the (**A** and **C**) P1-P2 (RdRp) and the (**C** and **D**) P3 (CP) genes of the poleroviruses identified in this study. Labels to the left of the figures indicate the amino acid sequences used for comparison. Numbers below the tree branches indicate the amino acid substitutions per site, and numbers above indicate the bootstrap support values; bootstrap values below 50% are not shown (trees were calculated using 1000 bootstrap replicates). Species demarcation is > 10% difference in aa identity of any protein. Isolates of the putative new recombinant polerovirus identified in this study, PaPV, are highlighted in red. References sequences are indicated by their GenBank accession numbers to the right of the virus label; accession numbers for the viruses identified in this study are listed in Table 4. Par_ and Car_ indicate the sequences were retrieved from parsley and carrot samples, respectively. The scales to the right indicate the percent identity scores displayed in the matrix. TaPV, Trachyspermum ammi polerovirus; TorCLV, Torilis crimson leaf virus; CtRLV, carrot red leaf virus; PaPV, parsley polerovirus; FvPV, Foeniculum vulgare polerovirus; WCtRLV, wild carrot red leaf virus; BVG, barley virus G (outgroup).

Of particular interest are the phylogenetic and percent identity aa comparisons of the PaPV sequences identified in this study with those of the CaPV1, TaPV, and TorCLV reference sequences. In phylogenetic comparisons of the P1-P2 aa sequences, the PaPV1 sequences clustered in the same clade as the CaPV1 reference isolate (Figure 4A), and shared 92% aa ID with this virus (Figure 4B). TaPV was also grouped in the same taxonomic cluster as PaPV, but shared less aa ID (up to 88%). Conversely, the TorCLV sequences clustered in their own separate clade, and shared only 70% aa ID with the PaPV sequences, while the TorCLV sequences identified in this study shared 97% aa ID with the reference isolate.

Comparisons of the P3 aa sequences conversely placed the PaPV viral sequences in the same lineage as TorCLV (Figure 4C), which shared 98% - 100% aa ID with the TorCLV reference isolate and the isolates obtained in this study (Figure 4D). In contrast the PaPV sequences only shared up to 66% aa ID with the CaPV1 reference isolate, and up to 74% aa ID with the TaPV reference isolate, which were each grouped in completely separate clades (4c). Altogether, these results demonstrate that the 5′ and 3′ gene blocks of the PaPV sequences vary dramatically in their respective lineages, with the P3 (CP) aa sequence from the 3′ gene block being nearly identical to that of TorCLV, while the P1-P2 aa sequence from the 5′ gene block likely originated from CaPV1. With regard to the other polerovirus sequences included in the analysis, these viruses all clustered in the same lineages as their respective reference isolates and shared ≥ 90% identity with them.

### Recombination analysis of PaPV

**FIGURE 5:**
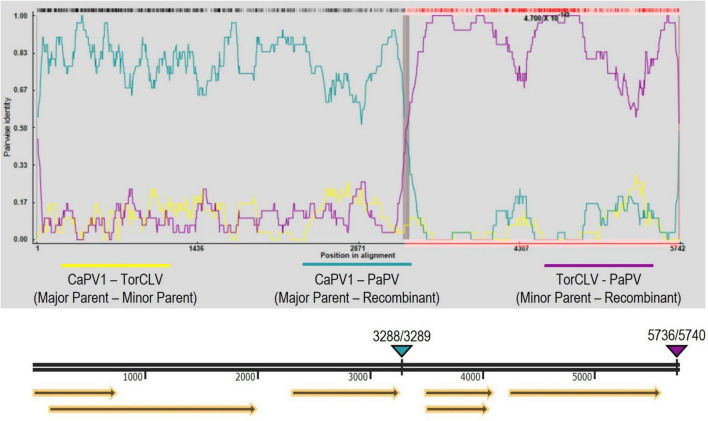
Recombination analysis of the putative new recombinant polerovirus PaPV. RDP analysis of CaPV1, TorCLV, and PaPV viral sequences. Yellow lines indicate pairwise nt comparisons across the entire length of the genomes of CaPV1 and TorCLV, teal lines indicate pairwise comparisons between CaPV1 and PaPV1, and magenta lines indicate pairwise comparisons between TorCLV and PaPV. The double black line beneath the recombination graph indicates the nt positions in the viral genome, yellow arrows beneath this line indicate the predicted reading frames, and the teal and magenta triangles above the line indicate the nt positions of the beginning and ending breakpoints in the viral genome. Average *P*-values for each of the recombination analysis methods performed by the RPD4 software are listed in Supplementary Table 4.

Recombination analysis of the two PaPV isolates (referenced as PaPV_1 and PaPV_2) and the CaPV and TorCLV reference genomes was performed using RDP4 software, using the complete suite of testing options available—RDP, GENECONV, BootScan, MaxChi, Chimaera, SiScan, 3Seq, LARD, and Phylpro. A recombination event was detected by all but one of the tests employed (Phylpro), with predicted *P*-values ranging from 4.440 x 10^–16^−8.567 x 10^–254^ (Supplementary Table 4). The major parent was predicted to be CaPV1 and the minor parent was predicted to be TorCLV. The predicted break beginning and end break points occurred at nt positions 3288 and 5736 for PaPV_1 (GenBank accession PP683457), and at nt positions 3289 and 5740 for PaPV_2 (GenBank accession PP683458) (Figure 5). It should be noted that is possible that CaPV1 and TorCLV may be recombinants of PaPV with other uncharacterized parental virus sequences.

### Umbravirus sequence identification

A total of six putative umbravirus sequences—CMoV, CMoMV, wild carrot mottle virus (WCMoV), parsley mottle virus, parsley mottle mimic virus, and tobacco bushy top virus (TBTV) were identified by RNAseq analysis, however parsley mottle virus, parsley mottle mimic virus, and TBTV were excluded from further analysis as they were poorly represented in the overall dataset, in that only a few (1–3) short (< 700 nt) contigs that were represented by < 1% of the total virus reads for the sample group and/or had < 100x genome coverage were retrieved for each of these viruses (Tables 2, 3). CMoV contigs were identified in seven of the 12 sequenced carrot samples, with nearly full length (∼4.2 kb) contigs recovered from four. Manual BLASTn searches of these contigs determined they shared > 97% nt identity with CMoV sequences in GenBank (LC434066.1, LC434065.1, LC434067.1, LC434068.1). According to the returned RNAseq results, CMoV was also identified in 12 of the 18 sequenced parsley samples and in the 2020 cilantro sample, however, manual BLASTn searches of these contigs revealed these contigs to be more closely related to a recently described umbravirus, Pastinaca umbravirus 1 (PasUV1; OL472236.1 and OL472237.1), sharing up to 81% nt sequence identity. CMoMV was identified in 10 of the parsley samples and six of the carrot samples, with nearly full length contigs (∼4.2 kb) from seven parsley and three carrot samples; these shared > 97% nt identity with CMoMV accessions (OQ993362.1, NC_001726.1, FJ188471.1). The third identified umbravirus, WCMoV, was found in a single flat leaf parsley sample and in eight carrot samples, with nearly full length (∼4.2 kb) contigs being recovered from five of these. Manual BLASTn analysis concordantly showed these shared 83% nt identity with the single WCMoV sequence in GenBank (LT615232.1).

### Phylogenetic and pairwise identity comparisons of identified umbravirus sequences

**FIGURE 6:**
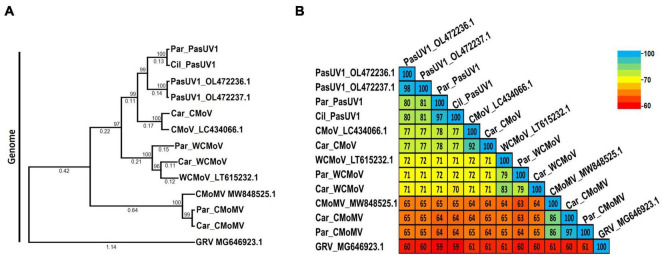
Phylogenetic tree and pairwise comparisons of umbravirus relationships. Depicted are the maximum likelihood phylogenetic tree (**A**) and percent pairwise identity matrix (**B**) constructed using nucleotide sequences of nearly complete umbravirus genomes. Numbers below the tree branches indicate the nucleotide substitutions per site, and numbers above indicate the bootstrap support values; bootstrap values below 50% are not shown (trees were calculated using 1000 bootstrap replicates). Species demarcation is > 70% nt identity across the entire genome. Reference sequences are indicated by their GenBank accession numbers to the right of the virus name; accession numbers for the viruses identified in this study are listed in Table 4. Par_ and Car_ indicate the sequences were retrieved from parsley and carrot samples, respectively. CMoV, carrot mottle virus; CMoMV, carrot mottle mimic virus; WCMoV, wild carrot mild virus; PasUV1, Pastinaca umbravirus 1 (PasUV1); GRV, groundnut rosette virus (outgroup).

According to the ICTV, the current species demarcation for umbravirus species is > 70% shared nt identity of the entire genome. Maximum likelihood phylogenetic and pairwise identity analyses were conducted using nearly full length genome sequences of the umbraviruses identified in this study to determine their relationships; groundnut rosette virus (GRV; GenBank accession MG646923.1) was used as an outgroup. The typical umbraviruses already known to be associated with CMD disease—CMoV and CMoMV—clustered in distinct clades along with their respective reference isolate sequences (Figures 6A, B). The CMoV and CMoMV sequences identified in this study shared 96% and 95% nt identity with their respective reference isolates (CMoV: LT615232.1; CMoMV: NC_001726.1). At the nt level, the WCMoV isolates obtained from parsley and carrot samples characterized in this study shared 79% and 83% nt identity, respectively, with the reference isolate (LT615232.1), and 79% nt identity with each other. The PasUV1 isolates from parsley and cilantro samples shared 80% - 81% nt identity with the refence isolates (OL472236.1, OL472237.1) and 97% nt identity with each other. Unexpectedly, however, the WCMoV and PasUV1 sequences (for both the reference isolates and those obtained in this study) shared ≥ 70% nt with each other, suggesting these viruses are not distinct species. Additionally, CMoV sequences shared ≥ 71% nt identity with WCMoV and ≥ 77% nt identity with PasUV1 (Figure 6B), which do not meet the species demarcation criteria umbraviruses, suggesting these umbraviruses may actually be divergent isolates of CMoV rather than distinct species. Phylogenetic comparisons of the near full length genome sequences of PasUV1 and WCMoV isolates placed each of these viruses in their own distinct subclades that grouped into a larger clade with CMoV, with CMoV being more closely related to PasUV1 than WCMoV (Figure 6A).

### tlaRNA sequence identification

Six different tlaRNAs were identified by the initial RNAseq analysis (Tables 2, 3). Manual BLASTn analysis of the retrieved contigs confirmed the presence of the following CtRLVaRNAs: CtRLVaRNAs a25, alpha, beta, gamma, and sigma. Additionally, another recently discovered tlaRNA, Arracacha latent virus E associated RNA (ALVEaRNA) was also identified and appeared to be the most abundant tlaRNA in terms of the number of samples in which it was found—four parsley samples and one carrot sample. Of the CtRLVaRNAs, a25, alpha, beta, and gamma were found exclusively in the carrot samples, with nearly full length contigs being retrieved for all. CtRLVaRNA sigma was found exclusively in four separate parsley samples, each yielding nearly full length contigs.

### Phylogenetic and pairwise identity comparisons of identified tlaRNA sequences

**FIGURE 7:**
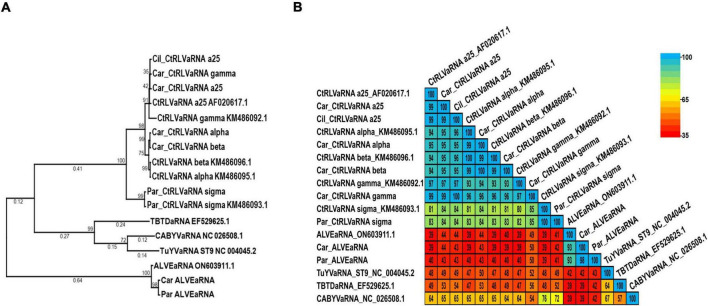
Phylogenetic trees and average percent pairwise matrices depicting relationships of the tlaRNA sequences found in this study. Depicted are a maximum likelihood phylogenetic tree (**A**) and percent pairwise identity matrix (**B**) constructed using the P1a+P1b RdRp translated amino acid sequences of the tombusvirus-like associated RNAs (tlaRNAs) identified in this study. Numbers below the tree branches indicate the amino acid substitutions per site, and numbers above the lines indicate the bootstrap support values; bootstrap values below 50% are not shown (trees were calculated using 1000 bootstrap replicates). No species demarcation criteria have been established for tlaRNAs. References sequences are indicated by their GenBank accession numbers to the right of the virus label; accession numbers for the viruses identified in this study are listed in Table 4. CtRLVaRNA, carrot red leaf virus associated RNA. ALVEaRNA, arracacha latent virus E associated RNA. Outgroup sequences, TuYVaRNA ST9, turnip yellows virus aRNA ST9 TBTDaRNA, tobacco busy top disease aRNA; CABYVaRNA, cucurbit aphid borne yellows virus aRNA.

As tlaRNAs remain formally unclassified, there are no specified species demarcation criteria against which to compare them, nonetheless we conducted phylogenetic and pairwise comparisons of the tlaRNAs found in this study using nearly complete P1a+P1b (RdRp) translated aa sequences; the turnip yellows virus (TuYV) ST9 isolate aRNA (TuYVaRNA ST9; NC_004045.2), tobacco bushy top disease (TBTD) aRNA (EF529625.1), and cucurbit aphid borne yellows virus (CABYV) aRNA (NC_026508.1) were used as outgroups. Among the tlaRNAs found in this study, the CtRLVaRNAs a25, alpha, beta, gamma clustered in the same clade and shared ≥ 93% aa identity (Figures 7A, B). CtRLVaRNA sigma isolates clustered within their own clade adjacent to the larger CtRLVaRNA clade, with the isolates in this study sharing 100% identity amongst themselves and with the reference isolate (KM486093.1), and 81% - 85% aa identity with the other CtRLVaRNAs. Interestingly, the ALVEaRNA isolates obtained in this study were farther removed from the CtRLVaRNAs than the outgroup tlaRNAs used in this study, sharing only 39% - 50% aa identity with either the CtRLVaRNAs or outgroup tlaRNAs.

### RT-PCR and whole plasmid nanopore sequencing validation of RNAseq results

Using primers designed for the specific detection of each of the poleroviruses, umbraviruses, tlaRNA Sigma and ALVEaRNA, and primers designed for the general detection of the other CtRLVaRNAs identified in this study we were able amplify by RT-PCR nearly full length amplicons of each of these viruses and tlaRNAs, and the cloned sequences were confirmed by nanopore sequencing. For polerovirus detection, we recovered ∼4.6 kb long amplicons of TorCLV, a ∼5.4 kb amplicon of CtRLV, a ∼4.3 kb amplicon of FvPV, and ∼4.6 – ∼4.7 kb sequences of the putatively novel PaPV recombinant polerovirus. For the umbraviruses, we obtained ∼3.9 kb amplicons of CMoMV from both parsley and carrot samples, a ∼4.1kb amplicon of CMoV, ∼4 kb long amplicons of PasUV1 from parsley, and ∼4 kb long amplicons of WCMoV. For the tlaRNAs, we retrieved ∼ 2.3 kb long amplicons of ALVEaRNA from both parsley and carrot samples, an ∼2.8 kb amplicon of CtRLVaRNA sigma, and ∼2.8 kb long amplicons of both CtRLVaRNAs a25 and alpha from carrot and cilantro samples. RT-PCR gel images are depicted in Supplementary Figure 2 and these results are summarized in Supplementary Table 2.

## Discussion

In this work, multiple recently described emergent polerovirus and umbravirus species, and one emergent tlaRNA species were identified for the first time in the U.S. using high through put sequencing. In addition to the typical viruses and tlaRNAs known to cause CMD and to occur in the U.S.—the polerovirus carrot red leaf virus (CtRLV), the umbraviruses carrot mottle virus (CMoV) and carrot mottle mimic virus (CMoMV), and CtRLVaRNAs (CtRLVaRNAs)—we identified the emergent poleroviruses Torilis crimson leaf virus (TorCLV) and Foeniculum vulgare polerovirus (FvPV), two potentially divergent, strains of CMoV, which we refer to by the names given them in published reports—Pastinaca umbravirus 1 (PasUV1) and wild carrot mottle virus (WCMoV), and the tlaRNA, arracacha latent virus E associated RNA (ALVEaRNA), none of which have been previously identified in the U.S. Lastly, but of particular interest, we discovered a putative new polerovirus that appears to be a recombinant of CaPV1 and TorCLV, for which we propose the name parsley polerovirus (PaPV).

Of the poleroviruses identified in this study, CtRLV was exclusively found in carrot samples, FvPV was identified in cilantro, TorCLV was identified in both parsley and cilantro, and PaPV was found exclusively in parsley. While a full-length genome sequence of TorCLV has been deposited in Genbank, there currently exist no published reports of this virus, and as such little is known about its biology, distribution, effects on symptom development, or what sort of risk, if any, it may pose to the production of economically important crop plants. According to the details included in the GenBank accession, this virus was first isolated in Greece from cultivated and weedy apiaceous plant samples. Given the name, Torilis crimson leaf virus, this virus was likely isolated from a plant in the genus *Torilis*, which are non-cultivated apiaceous plants broadly referred to as hedge parsleys, that are native to Northern Africa and Eurasia but have been spread to other countries, including the U.S.^3^ This report therefore adds to the limited information we have about this virus, expanding information on its host range to both parsley (*Petroselinum crispum*) and cilantro (*Coriandrum sativum*), and implicating it as a potential causative agent of CMD-like symptom development in these hosts.

FvPV was previously identified in an exploratory study in which publicly available transcriptomes from a variety of plant species available in the NCBI Transcriptome Shotgun Assembly (TSA) Sequence Database were bioinformatically mined for putative novel polerovirus sequences (Kavi et al., 2022). Partial (∼4.3 kb and ∼1.4 kb long) FvPV sequences were recovered from transcriptomes of *Foeniculum vulgare* (fennel) leaf samples that had been collected in Italy. The authors note that FvPV shared 88.5% - 89.1% nt sequence identity with partial RdRp coding sequences designated as belonging to Fennel motley virus (FMV), which would explain our initial detection of FMV in our preliminary RT-PCR diagnostic assays. Our finding of FvPV therefore represents the first report of this virus in cilantro as well as in the U.S.

CaPV1 was recently identified in carrot samples collected from fields in France and Spain (Schönegger et al., 2022). While we did not identify this virus in our samples, phylogenetic, pairwise identity, and recombination analyses revealed this virus to be the major parent of the putatively new recombinant polerovirus identified in this study, PaPV, with which it shared ∼92% identity of the RdRp translated aa sequence in the 5′ gene block (Figure 4B). TorCLV was identified as the minor parent of PaPV, sharing > 98% identity of the CP translated aa sequence in the 3′ gene block. While intriguing, this finding is not necessarily surprising as the intergenic region between the ORF2 RdRp gene and the ORF3 CP gene is a known recombination hotspot in poleroviruses, and there exist numerous reports of other recombinant poleroviruses for which the 5′ and 3′ gene blocks appear to have been acquired from different parent polerovirus species (Knierim et al., 2010; LaTourrette et al., 2021; Schönegger et al., 2022).

Of the umbraviruses identified in this study, CMoV was found exclusively in carrot samples, while CMoMV was found in both carrot and parsley samples. Sequences of two recently reported umbraviruses—WCMoV and PasUV1—were also identified in this study. WCMoV was found in multiple carrot samples and a single parsley sample; like TorCLV, no published reports of this virus currently exist, excluding the GenBank accession of the full length WCMoV genomic sequence. In our own analyses, we found that WCMoV shares > 70% nt identity of the nearly complete genome sequence with CMoV, suggesting that it may not be a distinct species but rather a divergent strain of CMoV (Figure 6B). PasUV1 was also identified in both parsley and cilantro samples. PasUV1 was recently identified in *Pastinaca sativa* (parsnip) plants, as part of a study in which field and greenhouse grown tomato plants from Slovenia, along with weeds found in the surrounding area, were surveyed for viruses by RNA sequencing (Rivarez et al., 2023). Based on phylogenetic and pairwise identity analyses using translated aa sequences of the RdRp, the authors proposed it to be a new umbravirus. In our own analyses, we found PasUV1 shared ≥ 77% nt identity with CMoV isolates, well above the 70% threshold (Figure 6B), again suggesting this virus may be a divergent strain of CMoV rather than a distinct species. Another metric used for discriminating umbravirus species is host range, which we could not evaluate in this study. As such, comparative host range studies of CMoV, WCMoV, and PasUV1 isolates could more precisely determine whether these are distinct species.

Among the tlaRNAs found, a multitude of CtRLVaRNAs—a25, alpha, beta, and gamma—were found exclusively in carrot samples, whereas another CtRLVaRNA, sigma, was found exclusively in parsley. Lastly, a recently described tlaRNA, ALVEaRNA, was found both in parsley and carrot samples. ALVEaRNA was first identified in arracacha (*Arracacia xanthorrhiza*) plants—a type of starchy root vegetable that is widely cultivated and eaten throughout South America—from Peru, in association with an enamovirus (family *Solemoviridae*) designated Arracacha latent virus E (De Souza et al., 2021). This tlaRNA has subsequently been identified in cultivated carrots in France and Spain, in association with CtRLV, indicating that it’s associations with a helper virus likely lack specificity (Schönegger et al., 2022) and in composite weed samples from Slovenia, in which the polerovirus Barley virus G was also found, although it can’t be said if they were isolated from the same plant (Rivarez et al., 2023). This is the first report of ALVEaRNA being found in parsley plants and in association with yet another potential helper polerovirus (TorCLV), and it’s first finding in the U.S.

Future research aimed at identifying the aphid vector(s) of the emergent poleroviruses TorCLV, FvPV, and PaPV found in this study could provide valuable insights into the potential host range overlap and epidemiological implications of the recent discovery of these viruses. The polerovirus historically associated with CMD, CtRLV, is vectored by the carrot-willow aphid *C. aegopodii*, which is known to feed on plants belonging to at least 10 different families, though they are most often found colonizing plants in the family Apiaceae, such as carrot, cilantro, and parsley which serve as secondary hosts, though its primary host plants belong to the willow family, Salicaceae (Favret and Miller, 2012). It would be of great interest to determine if *C. aegopodii* could likewise vector these emergent viruses, or if they have alternative aphid vectors with similar or differing host ranges. It should be noted that *Dysaphis apifolia* (Hawthorne-parsley) aphids were present on the 2020 cilantro sample used in this study, making this aphid a candidate vector of TorCLV, as well as PaPV, given the nearly identical aa sequences of their capsid proteins. In addition to identifying aphid vectors of the poleroviruses described here, it would be intriguing to examine the host ranges of each of the poleroviruses, umbraviruses, and tlaRNAs as well, and determine how those host ranges overlap for both the viruses as well as the aphid vectors. Interactions between poleroviruses, umbraviruses and tlaRNAs can be somewhat promiscuous in that tlaRNAs and umbraviruses have the potential to be non-specifically encapsidated by capsid proteins of different polerovirus species (Erickson and Falk, 2023). As such, overlaps in compatible vectors and plant hosts of these viruses could result in the emergence of additional recombinant poleroviruses as well as novel compatible polerovirus, umbravirus, and tlaRNA combinations that could be spread to naïve hosts.

The findings of this study not only add to the growing body of literature on the deployment of high throughput sequencing techniques for the detection and identification of emergent plant viruses and tlaRNAs, they also expand the known number of virus species and combinations of viruses and tlaRNAs associated with CMD and their respective hosts plants. This work highlights the plasticity of these polerovirus-umbravirus-tlaRNA interactions, the outcomes of which could have significant epidemiological implications for crop production.

## Data availability statement

The datasets presented in this study can be found in online repositories. The names of the repository/repositories and accession number(s) can be found in the article/Supplementary material.

## Author contributions

AE: Conceptualization, Data curation, Formal analysis, Investigation, Methodology, Validation, Writing–original draft, Writing–review and editing. AH: Data curation, Formal analysis, Software, Writing–review and editing. CC: Data curation, Formal analysis, Software, Writing–review and editing. BF: Resources, Writing–review and editing. Y-WK: Resources, Writing–review and editing.

## Footnotes

1
www.snapgene.com

2
https://inkscape.org/

3
www.itis.gov

## Supplementary material

Supplementary Figure 1 Integrative genomics viewer (IGV) analysis to look for contamination by host RNAs. Graph of RNAseq reads mapped back to a representative viral contig obtained in this study by RNAseq analysis. While viral reads can be seen to overlap across the entire viral contig, host reads occurring at the extreme 5′ and 3′ ends of the contig do not overlap with viral sequences.

Supplementary Figure 2 Depicted are 1% agarose gels displaying nearly full length RT-PCR amplicons of each of the viruses identified in this study. Numbers above the gels indicate the individual plant samples that were tested, numbers below the gels indicate the sample groups to which the samples belong. (**A**) Poleroviruses: CtRLV, carrot red leaf virus; PaPV, parsley polerovirus; TorCLV, Torilis crimson leaf virus; CaPV1, carrot polerovirus 1. (**B**) Umbraviruses: CMoV, carrot mottle virus; CMoMV, carrot mottle mimic virus; PasUV1, Pastinaca umbravirus 1; WCMoV, wild carrot mottle virus. (**C**) Tombusvirus-like associated RNAs: CtRLVaRNA, carrot red leaf virus associated RNA; ALVEaRNA, arracacha latent virus E associated RNA. Molecular weight marker: 1 kb plus DNA marker (Invitrogen).
