# Supplementary material for: Identification of a novel recombinant polerovirus and other emergent viruses and tombusvirus-like associated RNA species associated with carrot motley dwarf disease in the United States
Source: Front Microbiol. 2024 Jul 26;15:1430445. doi: 10.3389/fmicb.2024.1430445 (PMC11314183; doi:10.3389/fmicb.2024.1430445)
Supplement: Supplementary file 1 [file Data_Sheet_1.pdf]

Table S1: List of primers used in this study, along with the annealing temperatures, expected product sizes, and relevant citations.

	Primer name	Sequence	Annealing	Product	References
**Diagnostics**	AR3F	CAGATGAATTTCTGGCGTGCTA	56	650 bp	Campbell et al., 2020
	AR5R	TCCACCATCAACACYTGRTCRTCTCCRTCRTT*			
	Umbra-NNS	TGGWGTICACAACAACTC*	48	408 bp	Vercruysse et al., 2000
	Umbra-IBS	AAGGCTTTGTACAACATTGG			
	CtRLV-1	GAGGTGAGAAATCGCYTGAC	59	211 bp	
	CtRLV-2	MGGCGCCACARTGATAGG*			
	PolGen RT3	ACCTCGACTTTDA*	*For RT: 45	593 bp	Lotos et al., 2014
	PolGenUp2	GATGARGGTCGYTACCG*	58, 54, 52 (ramp down)		
	PolGenDown2	ACCTCGACTTTRAARCC*			
**RNAseq validation**	CtRLV_F	ATACACCACGTGCTTGCT	50	~5.4 kb	Designed for this study
	CtRLV_R2	GGAACTAGTTGTGCTGCCGCTTAGC			
	TorCLV_F	CGAGGAAGATATATGCAGCTTGAG	52	~4.6 kb	
	TorCLV_R2	CTGCCCACTGGCTTAATGAGG			
	FvPV_F2	CGGGCATAAAGATCATATTTCAGCTTGC	55	~4.3 kb	
	FvPV_R2	GCTCCCCATTCTATCTTTTTATCAGTATAACCTCC			
	PaPV1_F	AGGCACTTCTCTGTGGGAGC	55	~4.6 kb	
	PaPV1_R	TGTTGGAACCGGCGTTTTCC			
	CMoV_F	AGCACTTAGATTCCCAGTTTAGC	51	~4.1 kb	
	CMoV_R	TTGAGGCGGGCTTTTACTGT			
	CMoMV_F	ATGTGTGCATGGTACGAGGG	53	~3.9 kb	
	CMoMV_R	CTTGGGCTGTTACTCCTCAACC			
	PasUV1_F	TGTCCACCGCTCTCTCCAC	53	~3.9 kb	
	PasUV1_R	CCTGCAGCTCAGGTTGGAT			
	WCMoV_F	TTGGTACTCCTGTAACGCGG	53	~3.9 kb	
	WCMoV_R	AGACCTTGTTTTAAGGACAAGGATCC			
	Sigma_F	TTAGCAACCGCGGGAAAAATTTCC	55	~2.8 kb	
	Sigma_R	TCCGAAAGGATATGTTGGCTAGTCAG			
	ALVEaRNA_F	ACCATCATAGCCAGCATTCGTGG	55	~2.3 kb	
	ALVEaRNA_R	GCGCATTATGGACACGTTGC			
	GenaRNA_F	CCGAAAGGATAAGTTGCCACACGA	55	~2.8 kb	
	GenaRNA_R	GTCGCTAGTGGAAACCCAGC			
**RACE adapter primers**	5’ Anchor adaptor	GGCCACGCGTCGACTAGTACGGGIIGGGIIGGGIIG*	55	~500 bp	
	Abridged adaptor	GGCCACGCGTCGACTAGTAC			
	3’ Anchor adaptor	GGCCACGCGTCGACTAGTACTTTTTTTTTTTTTTTTT			
**RACE gene specific primers**	5’PaPV_GSP-R1	CAAGTATCCAAAGGGATTGCTCGG	55	~500 bp	
	5'PaPV_GSP-R2	TCAGAGCGCTCAAGGGTTG		~400 bp	
	3'PaPV_GSP-F1	CTTTCTCACAGGTCCTCCTGTACAG		~500 bp	
	3'PaPV_GSP-F2	ACAGTTATTCTAGACGAGCATCTGG		~400 bp	

	5'Foen_GSP-R1	CTGAGCAAGGTTAAGAACGCAG		~500 bp	
	5'Foen_GSP-R2	AGCTTGAACGTCATCAGCACCA		~400 bp	
	Foen_3'RACE_F1	CTATAGATGAGGTGCAGGCGCAG		~500 bp	
	Foen_3'RACE_F2	CGGATGATATGGATATTCCTGATTCTC		~400 bp	

* I (inosine) base pairs with A, C, and T nucleotides; D pairs with G, A, and T nucleotides; M pairs with A and C nucleotides; R base pairs with A and G nucleotides: W pairs with A and T nucleotides; Y pairs with C and T nucleotides.

Table S2: Listed are details about the cloned sequences submitted for nanopore sequencing to validate the results obtained by RNAseq, including the number of cloned sequences, the sequence lengths, and the names and percent shared sequence identity with reference isolates.

Host	Viruses		No. plasmids sequenced	length (nt)	% identity with reference isolate(s)	Reference isolate(s)	Reference accession(s)
Carrot	Polerovirus	CtRLV	1	5414	98.65	CtRLV	LC434061.1
Parsley		TorCLV	3	3075 - 4620	94.22 - 96.42	TorCLV	LT615235.1
Cilantro		FvPV	3	4320 - 4322	94.84	FvPV	BK059375.1
Parsley		PaPV	3	4629 - 4661	89.28 - 89.38	CaPV1	OP886450.1
					87.02 - 87.33	TorCLV	LT615235.1
Carrot	Umbravirus	CMoV	1	4068	97.98	CMoV	KF533714.1
Carrot		CMoMV	2	3906 - 3910	96.75 - 97.34	CMoMV	OQ993362.1
Parsley		CMoMV	2	3910	96.98 - 97.01	CMoMV	OQ993362.1
Carrot		WCMoV	3	3902 - 3905	83.61 - 83.89	WCMoV	LT615232.1
Parsley		WCMoV	1	4102	81.42	WCMoV	LT615232.1
Parsley		PasUV1	3	3942 - 3946	81.99 - 82.2	PasUV1	OL472237.1
Carrot	tlaRNA	CtRLVaRNA a25	1	2773	96.83 - 97.19	CtRLVaRNA a25	KM486095.1; NC_003871.1
Cilantro		CtRLVaRNA a25	3	2772 - 2774	97.30 - 97.37	CtRLVaRNA a25	NC_003871.1
Carrot		CtRLVaRNA alpha	2	2773 - 2774	96.76 - 96.83	CtRLVaRNA alpha	KM486095.1
Parsley		CtRLVaRNA sigma	1	2751	99.05	CtRLVaRNA sigma	KM486093.1
Carrot		ALVEaRNA	3	2266 - 2268	92.56 - 92.65	ALVEaRNA	ON603911.1
Parsley		ALVEaRNA	3	2266 - 2268	92.47 - 92.56	ALVEaRNA	ON603911.1

Table S3: Nucleotide or amino acid sequences and substitution models used for phylogenetic tree construction using MEGA11 software

Virus genus	Nucleotide (nt) or amino acid (aa) sequence used in the analysis	No. of positions in final alignment	Substitution model used	Figure in paper
Polerovirus	P1-P2; RdRp (aa)	985	JTT+G+I	4a
	P3; CP (aa)	196	WAG + G	4c
Umbravirus	Genome (nt)	3520	GTR+G	6a
**tlaRNAs**	P1a-P1b; RdRp (aa)	453	JTT+G	7a

Table S4: Analysis methods and associated average P-values for recombination analysis of PaPV sequences using RDP4 software

Method	Average P-value
RDP	2.259×10^{-134}
GENECONV	9.866×10^{-135}
BootScan	8.028×10^{-133}
MaxChi	2.199×10^{-56}
Chimaera	5.429×10^{-58}
SiScan	1.204×10^{-74}
3Seq	4.440×10^{-16}
LARD	8.567×10^{-254}

Table S5: Putative mycovirus and oomycete virus sequences identified by BLASTx and BLASTn analysis of RNAseq data

Family	Genus	Species	Contigs
Amalgaviridae	Unclassified	*Plasmopara viticola* lesion assoc. amalga like virus	2
Botourmiaviridae	*Botourmiavirus*	*Pestalotiopsis* botourmiavirus	1
	Magoulivirus	*Botrytis cinerea* ourmia-like virus	3
		Acremonium sclerotigenum ourmia-like virus	1
		Cladosporium cladosporioides ourmia-like virus	1
	Penoulivirus	*Plasmopara viticola lesion assoc. ourmia-like virus*	40
	Scleroulivirus	soybean leaf-assoc. ourmiavirus	4
	Unclassified	*Erysiphe necator* assoc. ourmia-like virus	56
Discoviridae	*Orthodiscovirus*	*Plasmopara viticola* lesion assoc. mycobunyavirales-	1
Fusariviridae	*Fusarivirus*	*Erysiphe necator* assoc. fusarivirus	4
		Fusarium poae fusarivirus	1
	Unclassified	*Pleospora typhicola* fusarivirus	1
Hypoviridae	*Alphahypovirus*	*Bipolaris oryzae* hypovirus	1
	Betahypovirus	*Fusarium oxysporum* dianthi hypovirus	1
Mitoviridae	*Duamitovirus*	*Beta vulgaris* mitovirus	1
	Mitovirus	*Erysiphe necator* assoc. mitovirus	41
		Plasmopara viticola lesion assoc. mitovirus	28
		Erysiphe necator mitovirus	21
		pea assoc. mitovirus	19
		Mitovirus sp.	4
		Erysiphales assoc. mitovirus	3
		soybean leaf-assoc. mitovirus	3
		Fusarium andiyazi mitovirus	2
		Colletotrichum higginsianum mitovirus	1
		Leptosphaeria biglobosa mitovirus	1
		Sclerotinia sclerotiorum mitovirus	1
	Unuamitovirus	*Alternaria arborescens* mitovirus	6
		Ophiostoma mitovirus	1
Mymonaviridae	*Sclerotimonavirus*	*Sclerotinia sclerotiorum* negative-stranded RNA virus	1
Narnaviridae	*Narnavirus*	*Erysiphe necator* assoc. narnavirus	9
		Plasmopara viticola lesion assoc. narnavirus	6
		Monilinia narnavirus	3
		Cladosporium tenuissimum narnavirus	1
		Sclerotinia sclerotiorum narnavirus	1
	Unclassified	*Erysiphales* narna-like virus	11
Partitiviridae	*Partitivirus*	*Fusarium solani* partitivirus	2
		Picoa juniperi partitivirus	1
Potyviridae	Unclassified	*Plasmopara viticola* lesion assoc. poty-like virus	1
Tombusviridae	*Tombusvirus*	Erysiphales assoc. tombus-like virus	1
		Erysiphe necator assoc. tombus-like virus	1
		Leveillula taurica assoc. tombus-like virus	1

Totiviridae	*Totivirus*	red clover powdery mildew-assoc. totivirus	8
		Erysiphe necator assoc. totivirus	2
		Xanthophyllomyces dendrorhous virus	2
		Puccinia striiformis totivirus	1
	Unclassified	*Plasmopara viticola* lesion assoc. toti	4
		Erysiphales assoc. toti-like virus	3
		Rhodosporidiobolus odoratus RNA virus	2
Unclassified	Unclassified	riboviria sp.	5
		Uromyces virus A	3
		Erysiphe necator assoc. virus	2
		Erysiphe necator assoc. abispo virus	1
		Erysiphe necator assoc. negative-stranded RNA virus	1
		Fusarium graminearum dsRNA mycovirus	1
		Macrophomina phaseolina fusagravirus	1
		Plasmopara viticola lesion assoc. ambiguivirus	1
		Sclerotinia sclerotiorum bunyavirus	1
Virgaviridae	*Tobamovirus*	*Plasmopara viticola* lesion assoc. tobamo-like virus	3
		Erysiphe necator assoc. tobamo-like virus	2
	unclassified	*Erysiphe necator* assoc. virga-like virus	6

Table S6: Putative arthropod and nematode virus sequences identified by BLASTx and BLASTn analysis of RNAseq data

Family	Genus	Species	No. of Contigs
Chuviridae	Unclassified	lishi spider Virus	2
Lispiviridae	*Nematovirus*	Wuchang romanomermis nematode virus	2
Mitoviridae	*Mitovirus*	*Thrips tabaci* associated mitovirus	3
Orthomyxoviridae	Unclassified	hemipteran orthomyxo-related virus	1
Phasmaviridae		Wuhan insect virus	5
Phenuiviridae		hymenopteran phenui-related virus	1
Solemoviridae		nelson sobemo-like virus	1
		Frankliniella occidentalis associated sobemo-like virus	1
Tombusviridae		crane fly tombus-like virus	1
Unclassified		brandeis virus	4
		Hubei levi-like virus	1
		Hubei Wuhan insect virus	1
		muthill virus	1
		Shahe levi-like virus	1
		barley aphid RNA virus	4
		gorebridge virus	2
		Hubei partiti-like virus	1
		Beihai narna-like virus	1
