# Supplementary figures and images for: Identification of a novel recombinant polerovirus and other emergent viruses and tombusvirus-like associated RNA species associated with carrot motley dwarf disease in the United States
Source: Front Microbiol. 2024 Jul 26;15:1430445. doi: 10.3389/fmicb.2024.1430445 (PMC11314183; doi:10.3389/fmicb.2024.1430445)

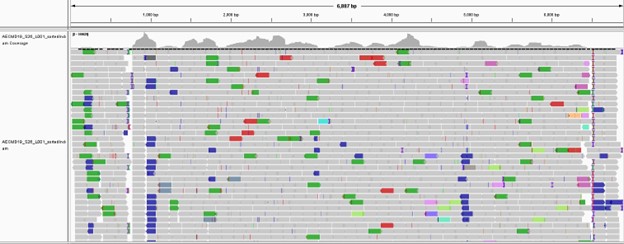

Supplement: Supplementary Figure 1 — Integrative genomics viewer (IGV) analysis to look for contamination by host RNAs. Graph of RNAseq reads mapped back to a representative viral contig obtained in this study by RNAseq analysis. While viral reads can be seen to overlap across the entire viral contig, host reads occurring at the extreme 5′ and 3′ ends of the contig do not overlap with viral sequences. [file Image_1.jpeg]

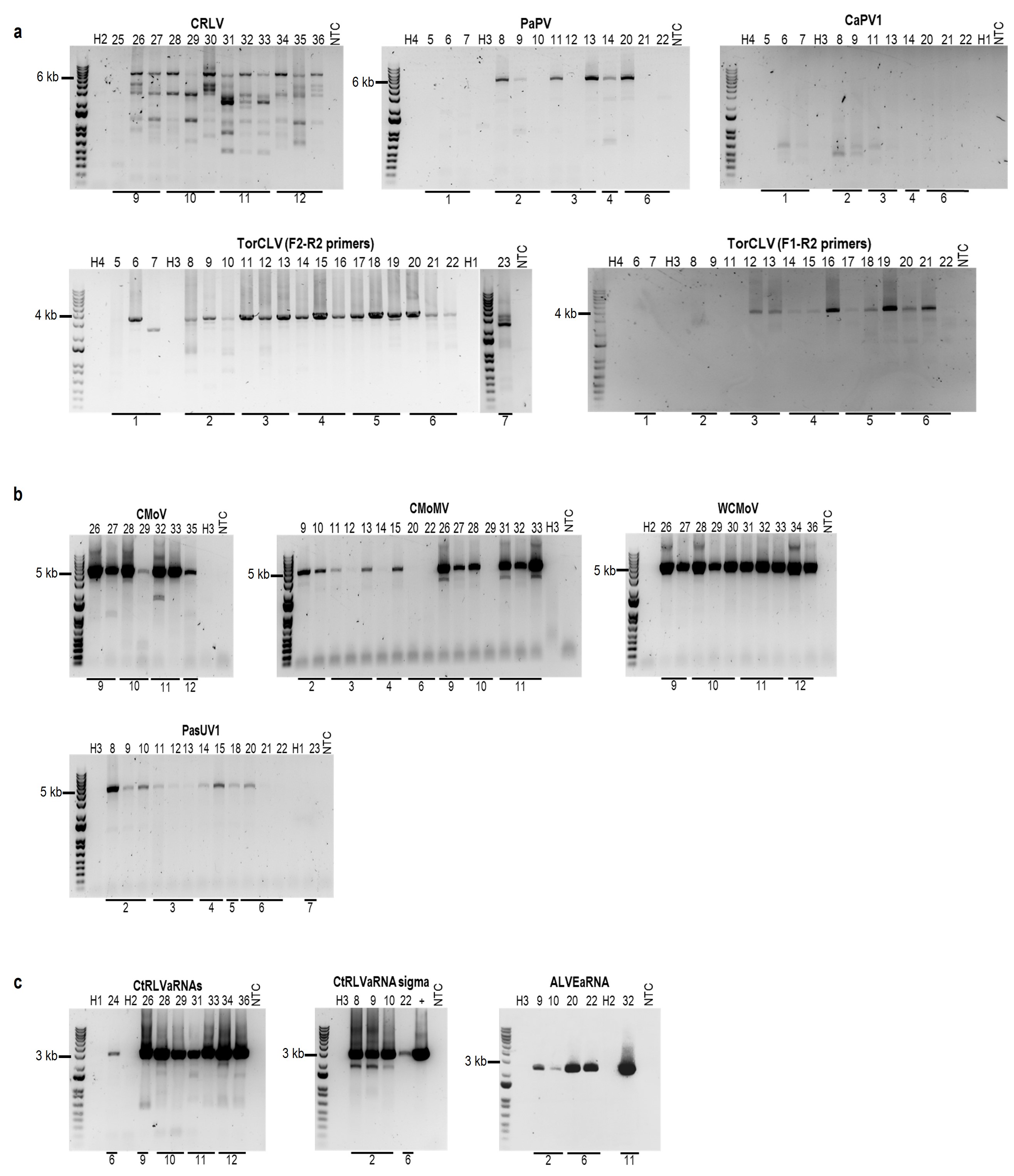

Supplement: Supplementary Figure 2 — Depicted are 1% agarose gels displaying nearly full length RT-PCR amplicons of each of the viruses identified in this study. Numbers above the gels indicate the individual plant samples that were tested, numbers below the gels indicate the sample groups to which the samples belong. (A) Poleroviruses: CtRLV, carrot red leaf virus; PaPV, parsley polerovirus; TorCLV, Torilis crimson leaf virus; CaPV1, carrot polerovirus 1. (B) Umbraviruses: CMoV, carrot mottle virus; CMoMV, carrot mottle mimic virus; PasUV1, Pastinaca umbravirus 1; WCMoV, wild carrot mottle virus. (C) Tombusvirus-like associated RNAs: CtRLVaRNA, carrot red leaf virus associated RNA; ALVEaRNA, arracacha latent virus E associated RNA. Molecular weight marker: 1 kb plus DNA marker (Invitrogen). [file Image_2.jpeg]
